# Modifiable motion graphics for capturing sensations

**DOI:** 10.1371/journal.pone.0229139

**Published:** 2020-02-24

**Authors:** Maria Galve Villa, Carsten D. Mørch, Thorvaldur S. Palsson, Shellie A. Boudreau

**Affiliations:** 1 Center for Neuroplasticity and Pain (CNAP), Center for Sensory-Motor Interaction (SMI)^®^, Department of Health Science and Technology, Faculty of Medicine, Aalborg University, Aalborg, Denmark; 2 Laboratory for Musculoskeletal Pain and Motor Control, Center for Sensory-Motor Interaction (SMI), Department of Health Science and Technology, Aalborg University, Aalborg, Denmark; University of Rome, ITALY

## Abstract

**Objective:**

The purpose of this study was to assess the relationship between an embodied sensory experience and the ability to translate the perception of this experience visually using modifiable motion graphics.

**Methods:**

A custom-designed software was developed to enable users to modify a motion graphic in real-time. The motion graphics were designed to depict realistic visualizations of pain quality descriptors, such as tingling and burning. Participants (N = 34) received an electrical stimulation protocol known to elicit sensations of tingling. The protocol consisted of eight stimulation intensities ranging from 2—6mA delivered, in a randomized fashion and repeated three times, to the index finger. Immediately after each stimulus, participants drew the area of the evoked sensation on a digital body chart of the hand. Participants then modified the motion graphic of tingling by adjusting two parameters, namely the speed (rate of dots disappearing and re-appearing) and density of these dots in the drawn area. Then, participants rated the perceived intensity and selected the most appropriate pain quality descriptor.

**Results:**

There was an increase in the area, density, and perceived intensity ratings as the electrical stimulation intensity increased (*P*<0.001). The density of the motion graphic, but not speed, correlated with perceived intensity ratings (0.69, *P*<0.001) and electrical stimulation intensities (0.63, *P*<0.01). The descriptor ‘tingling’ was predominantly selected in the range of 3–4.5mA and was often followed by ‘stabbing’ as the electrical intensity increased.

**Discussion:**

The motion graphic tested was perceived to reflect a tingling sensation, the stimulation protocol elicited a tingling sensation, and participants adjusted one of the two motion graphic features systematically. In conclusion, an embodied sensation, such as tingling, maybe visually represented similarly between individuals. These findings create research, clinical, and commercial opportunities that utilize psychophysics to explore, visualize, and quantify changes in embodied sensory experiences in response to known stimuli.

## Introduction

The field of psychophysics explores the transition from a physical stimulation to the perception of the evoked response. Additionally, psychophysical measures aim to scale or quantify an embodied sensory experience. For example, to perceive pain, the central nervous system must process and then interpret the stimulus (noxious or non-noxious) as painful. Typically, pain serves as warning a signal and demands our attention.

With regard to the perception of pain, the interpretation of the stimulus is influenced by genetic [1,2], pathological [3,4], cognitive [5], and psychological factors [6–8], making universal assessments of pain difficult. Pain intensity, quality descriptors, and pain location through the use of scales, questionnaires and drawings, respectively, are conventional approaches for measuring the pain experience in experimental and clinical settings [9]. Of all three approaches and despite known disadvantages [10], the pain experience is most often quantified as pain intensity and collapsed into a single number from 0 “no pain” to 10 “worst pain imaginable” [11]. Pain drawings are sometimes used to indicate the location of pain [12,13] and, more recently, to capture the extent (total area or number of pain sites) or widespreadness [14–20]. Pain quality descriptors such as burning, stabbing, or aching, help guide diagnosis by illuminating driving mechanisms of pain [9,21,22]. These pain quality descriptors are recognized as crucial indicators of symptom progression [9,23] and serve to differentiate between sources or types of pain, such as nociceptive and neuropathic pain [24–26].

The ability to associate a pain quality descriptor to a specific painful or non-painful sensation is limited by self-awareness, language skills, and prior experiences [27,28]. Despite these limitations, clinical assessment questionnaires, such as the McGill Pain Questionnaire (MPQ) or painDETECT [29–34], use pain quality descriptors routinely. These questionnaires use a series of pain descriptors to qualify the pain experience [35–37] and rate the pain descriptor intensity [26,38]. Additionally, psychometric scales used in experimental settings explore perception from a multidimensional perspective. For example, the individual-differences scaling model (INDSCAL) assesses the perceived pain quality (ranging from tingling to hammering), the pain quality intensity (ranging from slight to severe), and the emotional quality (ranging from comforting to upsetting) [39]. However, none of these psychometric tests can accurately quantify changes in embodied sensory experiences in response to known stimuli.

Abnormal or unusual non-painful sensations can accompany pain but may also occur before the onset of pain. For example, tingling is present in approximately 60% of patients with neuropathic pain and 16% of patients with non-neuropathic pain [30]. Moreover, tingling and numbness can occur during the early stages of peripheral neuropathy or a variation of Guillain-Barre syndrome [40,41]. Tingling may also result from ischemia of afferent neurons or altered central or peripheral neuronal activity [42–44]. Thus, non-painful sensations play an important role in the clinical presentation of some medical conditions. Therefore, communicating and quantifying changes in non-painful sensations is clinically relevant. However, pain and non-painful quality descriptors are difficult for patients, as the meaning of words can be ambiguous and differ across cultures [27,45]. Therefore, assessment tools that can go beyond the use of words to qualify and quantify changes in non-painful sensations, such as tingling or numbness, may lead to a better understanding of a patients’ condition, symptom progression, and underlying pathophysiology.

Currently, digital pain drawings use symbols and colours for the recording of pain qualities [19,46–52]. Symbols or colours provide an additional benefit of coding more information into the pain drawing but still require comprehensive language skills and prior experience. Alternatively, illustrations of pain qualities offer a means to explain or educate. Illustrations are static images and typically depict concepts of qualitative descriptors. For example, prickling is illustrated by a sewing needle pricking the skin and throbbing by a hammer over a body region. These illustrations aim to depict sensations that are inherently non-static. One could argue, these commonly used illustrations may not entirely reflect the actual sensation, be perceived as childish, and raises the risk belittling the significance of the patients’ symptom reports. Motion graphics, however, could build upon the benefits achieved with illustrations and enable a more realistic depiction of a sensation.

The purpose of this study was to assess the relationship between changes in an embodied sensory experience evoked by differing stimulus intensities as captured using motion graphics. More specifically, the relationship between perception and intensity of an electrically evoked tingling sensation and the ability to translate the perception visually using a modifiable motion graphic. This study assessed individual adjustments behaviour of a tingling motion graphic and the relation to the intensity of an electrical stimulus and the intensity of the evoked ‘tingling’ sensation. A secondary aim was to conduct semi-structured interviews to gain qualitative feedback about the motion graphic design and how to improve the representation of the embodied sensation evoked by the electrical stimulations.

## Materials and methods

### Participants

Thirty-four healthy participants (85% Caucasian, seven left-handed, 14 females, age range 21 to 32 years) were recruited through local social media groups and posters displayed on the university campus at Aalborg University (Denmark). Avoiding the word “tingling” during the recruitment and throughout the experimental session was essential to remove the possibility of selection bias when choosing verbal descriptors concerning the experimentally evoked tingling protocol. Thus, the word “sensation” replaced tingling when relevant.

Exclusion criteria included poor command of the English language, current or past history of chronic pain, or any condition that may affect sensory perception at the level of the fingertips, such as diabetes or neuropathies, scars, thick calluses, and injuries. Musicians have reduced sensitivity and thick calluses of the fingertips [53,54], and therefore, expert and novice musicians were excluded. Additionally, this study required the control of a computer mouse to adjust the motion graphics. Thus, right-handed and left-handed participants who regularly use their right-hand for controlling a computer were included.

The regional ethics committee in the North Denmark Region (N- 20150052) approved the experimental protocol. All participants gave written informed consent in accordance with the Declaration of Helsinki.

### Study design

**Fig 1:**
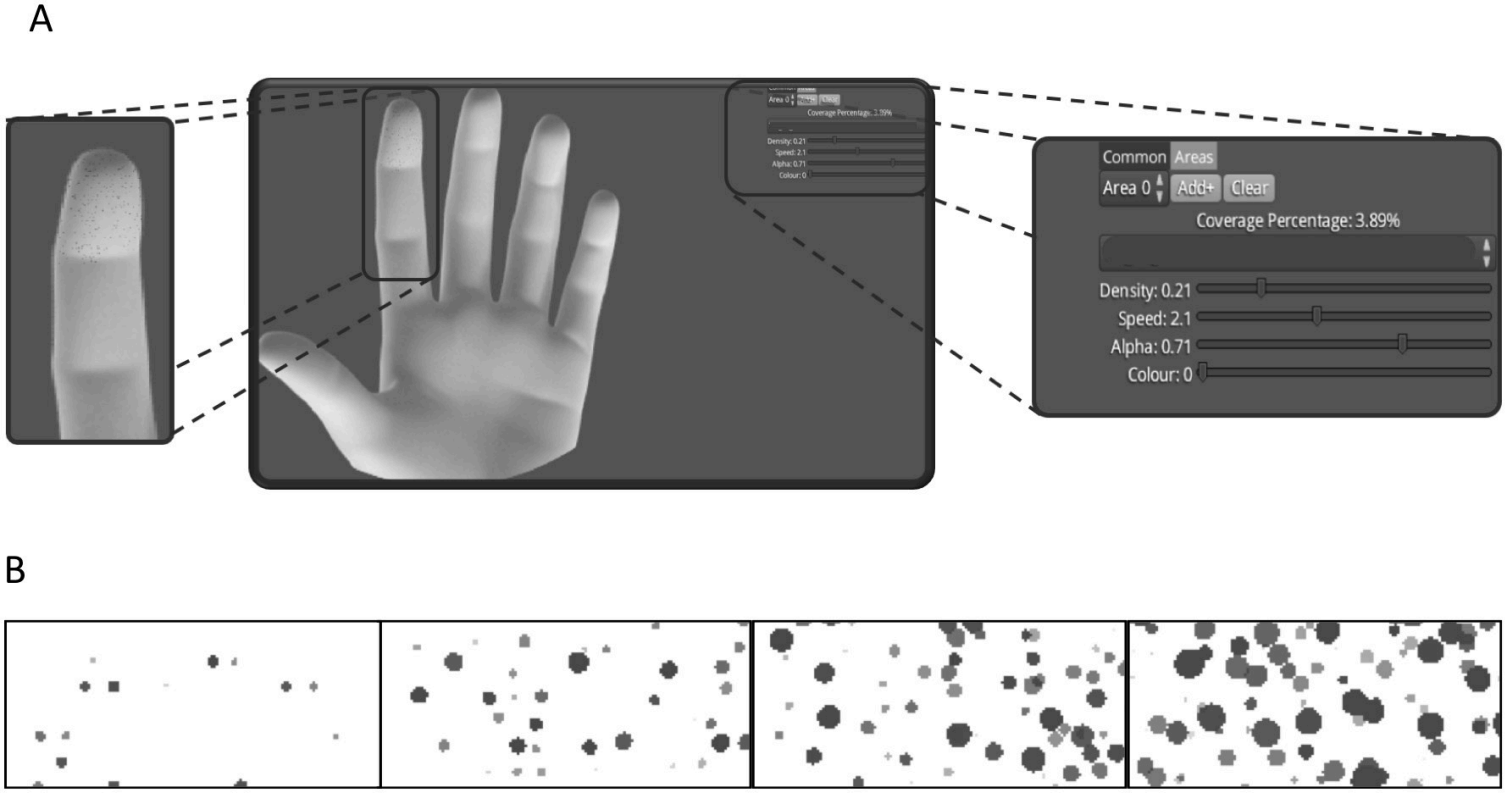
Screenshots of the motion graphic. (A) Dashboard display showing the digital body chart of the glabrous aspect of the hand showing a tingling motion graphic using the custom-designed software Animate Pain (Aalborg University, Denmark) with enlarged screenshot images of the static motion graphic on the drawn area of evoked sensation, as well as the density and speed VAS. (B) Static images of the motion graphic showing four of the variations of the density feature showing 0 (minimum), 0.2, 0.5, and 1 (maximal) values for density enlarged approximately 10x for clarity.

The study consisted of a single (1-hour) experimental session that included a familiarization protocol. A computer displaying a digital body chart of the hand and two visual analog scales (VAS) was placed directly in front of the participant. Participants modified a motion graphic (Fig 1) designed to depict a tingling sensation in response to transcutaneous electrical stimulation protocol known to elicit tingling sensations. The participants were unaware that the motion graphic was designed to depict tingling. More specifically, participants were instructed to modify the motion graphic to match the electrically evoked sensation using two graphic modifiers. To do this, participants adjusted one or both of the VAS that altered the density of dots (density) or the rate the dots appeared and re-appeared (speed). Following a range of electrical stimulations delivered to their index fingertip, with approximately 1-min interstimulus intervals, participants first drew the area of the evoked sensation on the hand using a computer mouse. They then modified the motion graphic to the best of their ability. Further, the participants rated the intensity of the stimulation and chose one of 12 pain descriptors that best described the evoked sensation. At the end of the experimental session, participants completed a semi-structured interview about the motion graphic design.

#### Protocol for electrically evoked tingling sensations

Transcutaneous electrical stimulations applied to the glabrous aspect of the finger-tip by way of surface electrodes (Neuroline 700, Ambu A/S, Denmark) was used to elicit a tingling sensation. The hand is a common body region in which individuals experience tingling sensations. Tingling sensations can occur due to a reduction in blood flow, for example, as a result of poor posture during sleep and are known to occur in association with neuropathic pain [55]. For this study, the index figure was chosen as a relevant and accessible test site. In a seated position, the participants’ left forearm rested on a table, with the palmar aspect facing upwards during the stimulus application. The skin of the left index finger was cleaned with water and dried before placing the surface electrodes. The electrodes were placed on the proximal and distal phalanges and connected to an isolated bipolar constant current stimulator (DS5, Digitimer Ltd, Hertfordshire, UK).

The protocol for electrically evoked tingling sensations consisted of 8 electrical stimulation intensities (2, 3, 3.5, 4, 4.5, 5, 5.5 and 6mA) with a constant number of bursts, frequency, duration of the burst and pulse width (1 burst, 250Hz, 4 seconds and 50μs, respectively). The electrical stimulation protocol was controlled by custom-made software *(Mr*. *Kick III v*. *3*.*0*, *Aalborg University*). The stimulation protocol was randomized and to circumvent a possible decline in memory between stimultions and motion graphic adjustments repeated three times, resulting in 24 stimulations per session. The data-collection for the 24 electrical stimulations lasted 30 minutes and consisted of an inter-stimulus interval of approximately 1-min. The protocol is unlikely to result in habituation due to the relatively long duration (1 min) of the inter-stimulus interval [56].

Pilot studies (N = 5) revealed that these electrical stimulation parameters evoked sensations that were perceived as tingling. These pilot sessions also revealed that four of the five participants were unable to perceive any differences in the sensation intensity, density, or speed between the 2mA and 2.5mA stimulation intensities. Therefore, the protocol incremented from 2mA to 3mA to minimize the number of electrical stimulations and statistical comparisons.

#### Familiarization protocol

Prior to initiating data collection, a familiarization protocol consisting of three randomly generated electrical stimulation intensities were delivered to the index distal phalanx. The familiarization protocol was given to (1) minimize nervousness related to receiving electrical stimulations, (2) to practice drawing the area of sensation with the mouse on the digital body chart of the hand and (3) to practice adjusting the motion graphic’s density and speed features using a hand-held mouse.

#### Motion graphic design

The design of the tingling motion graphic aimed for a realistic representation of a tingling sensation, as shown in Fig 1. The tingling motion graphic consists of small dots that appear, disappear, and reappear when an area of the perceived sensation is drawn on the body chart (Fig 1B). A custom-designed software application (*Animate Pain v1*.*0*, *Aalborg University*, *Denmark*) displays the motion graphic together with a high-resolution body chart of the glabrous aspect of a left hand on an interactive dashboard. The interactive dashboard enables modifications to the motion graphic on the body chart in real-time. In this study, two features of the motion graphic were adjusted in response to electrical stimulation by using a digital visual analog scale (VAS). Sliding the weights on the VAS modified the speed and density of the dots (S1 Multimedia file).

#### Recordings of the perceived descriptors, intensity location, and area of evoked sensation

Participants sat directly in front of the computer screen displaying the interactive dashboard with their right hand resting over a computer mouse. The left arm was supinated, with the elbow flexed and the hand resting on a table in a comfortable position, with the electrodes in place for the electrical stimulations. Prompting the left forearm with a towel minimized contact with the cold table surface and reduced pressure on the wrist joint. Participants were asked to draw the area of the electrically evoked sensation directly on the body chart using the computer mouse with their right hand. Once drawn, the motion graphic appeared.

The participants were instructed to adjust the speed and density using the two VAS, to the best of their ability, to create the most accurate representation of the electrically evoked sensation. Verbal instructions to the participant explained the purpose and function of the VAS for density and speed. Specifically, sliding the density weight to the right, increased the number of dots on the motion graphic. Sliding the speed weight to the right increased the rate of the dots disappearing. Meaning that increasing the speed reduced the amount of time the dots remained on display in the motion graphic. The density VAS ranged from 0.0–1.0 (dots per unit), and the speed VAS ranged from 0.0–5.0 (time to disappearance). The anchors accommodated a large range and, when adjusted, modified the shape language of the motion graphic, and, as such, the values are arbitrary units. No anchors were labelled on the VAS and were accurate to the 10th decimal. At the onset of the study, the density and speed VAS were preset to 1.0 and 0.5, respectively. The drawn area was automatically calculated in pixels and expressed as a percentage of the total body chart area. Once the participant modified the motion graphic, the area and VAS values were recorded and used for offline for statistical analyses.

After adjusting the motion graphic, participants rated the perceived intensity of the evoked sensation for each electrical stimulation on an NRS (0 = no sensation perceived; 10 = worst imaginable sensation of the quality perceived). Participants then selected a pain quality descriptor best describing the electrically evoked sensation. Participants were given the option to choose one word from a pre-defined list of 11 words selected from the short-form MPQ, as well as the descriptor “other” [38]: tingling, numbness, itchy, stabbing, drilling, sharp, spreading, dull, burning, warm, cooling, and other. The perceived intensity ratings and pain descriptors were recorded and stored offline for statistical analyses.

#### Participants’ feedback on the tingling motion graphic design

At the end of the session, an individual, semi-structured interview was carried out to determine the usability of the display panel and perceptions of the motion graphic as a starting point for visualizing a tingling sensation. The semi-structured interviews were conducted by the person (MVG) that administered the electrical stimulations. An interview guide was used, so the same topics were addressed for all the participants. The interview consisted of three open-ended questions “*do you think the motion graphic represented the sensations evoked by the stimulations*?”, “*how user-friendly did you find Animate Pain*?” and “*would you be able to make suggestions into how can Animate Pain be improved*?”. The interviews lasted 1–3 minutes. The answers were transcribed and coded. The responses were analyzed using the Grounded Theory analytic approach [57].

### Statistical analyses

Each electrical stimulation intensity was repeated three times. Thus, the geometrical mean (average of three) for each stimulation intensity was calculated for the density and speed values, as well as the associated perceived intensity ratings, and area of evoked sensation. Only data from electrical stimulations evoking a sensation were included. Histograms and Q-Q plots revealed a non-normal distribution of perceived intensity ratings (range 0–10), speed (range 0–5), density (range 0–1) values, and area (range 0–100%).

Friedman tests were used to determine differences in perceived intensity ratings, density and speed values, and size of the area of the evoked sensation among the different electrical stimulation intensities. Post-hoc analyses were performed using Wilcoxon signed-rank tests to locate the differences in perceived intensity ratings, evoked sensation area, graphic’s density, and speed values among the electrical stimulation intensities. A Bonferroni correction was applied for multiple comparisons. Furthermore, a graphical “trend” analysis using the first three (random) electrical stimulation ratings were performed to determine any evidence of a learning curve occurring by the repetition adjusting the motion graphic’s modifiers, as well as habituation or adaptation effects.

Spearman’s correlations were used to determine relationships between electrical stimulation intensity and the size of the evoked sensation area, perceived intensity ratings, and graphic density and speed. A Bonferroni correction was applied for multiple correlations.

#### Exploratory tingling and non-tingling data sub-analyses

Responses in the selection of the pain descriptor were divided between those participants who reported tingling and those who reported non-tingling sensations. Therefore, a tingling data-sub-set and a non-tingling data-subset were extracted. Planned exploratory sub-analysis on the perceived intensity ratings, density and speed features eliciting tingling and non-tingling sensations were performed to determine differences and similarities between tingling and non-tingling. Additionally, this sub-analysis explored the adjusting behaviour of the tingling sensations in response to a range of electrical stimulations. Spearman’s correlations were used to determine associations between changes in electrical stimulation intensity and the size of the evoked sensation area, perceived intensity ratings and density, and speed values of sensations specifically perceived as tingling and of those perceived as non-tingling.

#### Exploratory pain descriptor sub-analyses

A second planned exploratory sub-analysis was carried out to explore changes in the selection of tingling as a pain descriptors and the range of electrical stimulation intensities. A binary logistic regression was carried out to determine the influence on the stimulation intensity, as well as the repetition of the electrical stimulations, on the likelihood that participants would select tingling as their descriptor of choice. Furthermore, Kruskal-Wallis analyses were used to identify differences in density and perceived intensity ratings among the most common pain descriptors at specific electrical stimulations.

Statistical analyses were performed using SPSS 25 (*SPSS Statistics*, *2018*). Correlation coefficients, means and 95% confidence intervals (95% CI) are reported where relevant. Non-normally distributed data are presented as medians and IQ ranges. The error bars in the graphics represent the standard error of the mean (SEM). *P*-values of less than 0.05 were considered statistically significant. The post-hoc *P*-values were calculated and adjusted for multiple comparisons.

## Results

Two participants detected less than 50% of the total electrical stimulations, and their data were excluded from all analyses. The remaining thirty- two participants had a mean age of 28.13± 3.28 (7 left-handed, 13 females, 88% Caucasian). Eighty-two percent of the 2mA and 22% of the 3mA electrical stimulations failed to evoke any sensation. Therefore, the analysis included only responses from 3mA to 6mA.

### Perceived ratings of intensity, density, speed, and area

**Fig 2:**
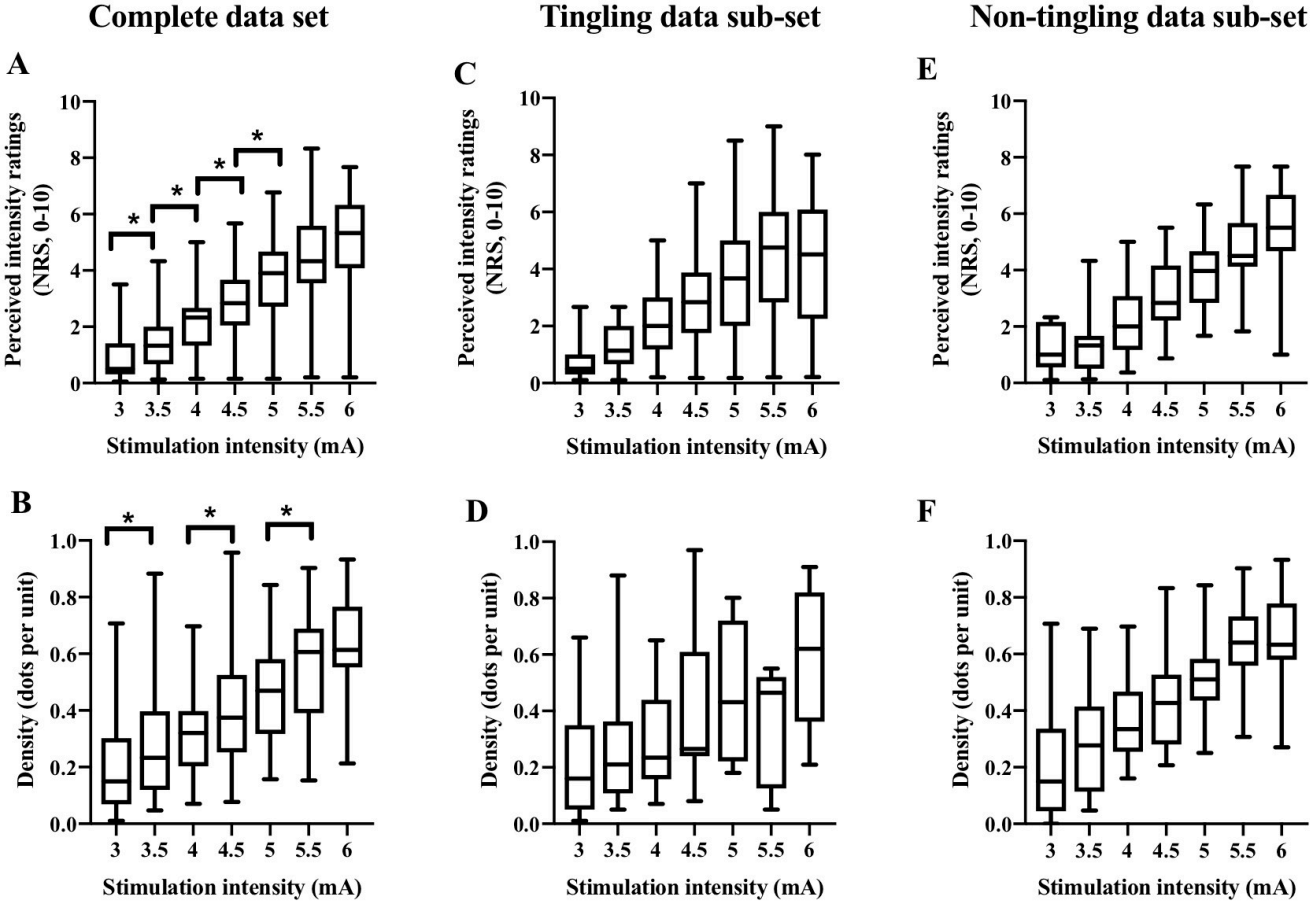
The relation between electrical stimulation intensity and perceived intensity ratings and density. Increases in perceived intensity ratings and density for the motion graphic occurred in associated with increases in electrical stimulation intensity for the complete data set (N = 641 perceived stimulations) (A, B), in the tingling data sub-set (N = 252 stimulations) (C, D) and the non-tingling data sub-set (N = 389 stimulations) (E, F). Significance adjusted for multiple correlations set at *P*<0.001. Box and whiskers represent the median (line), maximal, and minimal values.

**Fig 3:**
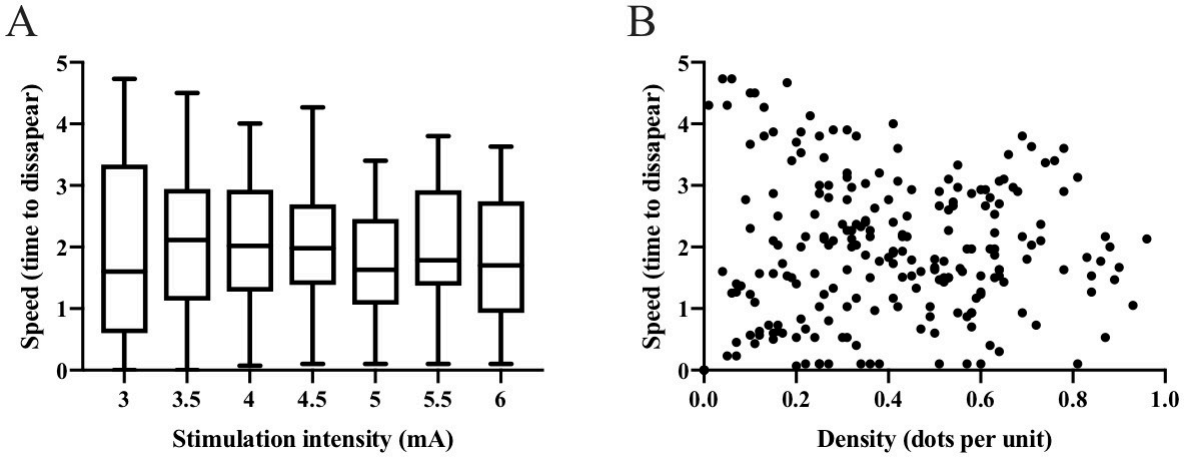
Correlation between density and speed. Speed values of the motion graphic showing no relation to the electrical stimulation intensity (A) or density (B) in the complete data set.

Friedman’s ANOVA revealed that the perceived intensity ratings, density, and speed values, as well as the evoked sensation area, differed with electrical stimulation intensity χ^2^(3) = 359.56 (*P*<0.001). A stepwise increase in perceived intensity ratings and density values occurred from 3mA to 5mA and 5.5mA, respectively, for the complete data set (*P* <0.001; Table 1, Fig 2A and 2B). Additionally, positive correlations were found between the perceived intensity ratings, the intensity of the electrical stimulations, and the density values in the complete data set (*P*<0.001, Table 2). There was no stepwise increase among the perceived intensity, density, and the stimulation intensity in the tingling and non-tingling data sub-sets (*P*>0.05, Fig 2B, 2C, 2D and 2E). No stepwise differences were evident in the area of the evoked sensation or the speed values, in response to the electrical stimulation increase in the complete data set (*P*>0.05). No relationship (*P*>0.05) was found between the speed and the density values (Fig 3), perceived intensity ratings, and the size of the evoked sensation area.

**Table 1:** Summary of the complete data set per electrical stimulation intensity.

Stim. intensity	Perceived intensity ratings (0–10)	Density (0–1)	Area (%)	Speed (0–5)
**3mA**	0.96 (0.63–1.29)	0.21 (0.14–0.28)	1.40 (1.01–1.78)	2.19 (1.63–2.75)
**3.5mA**	1.43 (1.11–1.75)*	0.28 (0.21–0.36)*	1.87 (1.35–2.39)*	2.15 (1.70–2.61)
**4mA**	2.11 (1.69–2.52)*	0.32 (0.27–0.38)	2.08 (1.62–2.54)	2.03 (1.61–2.45)
**4.5mA**	2.95 (2.44–3.46)*	0.41 (0.33–0.48)*	2.5 (2.05–2.95)	1.98 (1.62–2.35)
**5mA**	3.83 (3.28–4.39)*	0.46 (0.40–0.52)	2.74 (2.31–3.16)	1.79 (1.47–2.12)
**5.5mA**	4.52 (3.93–5.11)	0.55 (0.48–0.62)*	3.07 (2.56–3.58)	2.03 (1.67–2.38)
**6mA**	5.03 (4.39–5.67)	0.62 (0.56–0.69)	3.37 (2.94–3.79)*	1.86 (1.47–2.24)

Ratings of perceived intensity, area (expressed as a percentage of the body chart), and modifications to the density and speed values controlling the tingling motion graphic, in response to a range of transcutaneous electrical stimulation intensities applied to the index finger. Significance* adjusted for multiple correlations set at *P*<0.001 and listed for each additional increase. Data are presented as mean and 95% CI.

**Table 2:** Relationships among the electrical stimulation intensity, perceived ratings of intensity, density, speed, and area.

Parameters	Complete data set
**Stimulation intensity (mA)**	
Perceived intensity ratings (NRS, 0–10)	0.74* (54%)
Density (dots per unit)	0.63* (39%)
Evoked sensation area (%)	0.53* (25%)
**Perceived intensity ratings (NRS, 0–10)**	
Density (dots per unit)	0.69* (45%)
Evoked sensation area (%)	0.49* (17%)

Correlation coefficients and adjusted R-squared (Goodness-to-fit) values in brackets, for the complete data set. Significance* adjusted for multiple correlations set at *P*<0.001.

Post-hoc analyses on the first three random stimulations and the tingling data sub-set show that the perceived intensity ratings and density values have a similar graphical trend in response to an increase in electrical stimulation intensity, as compared to the complete data set (S1 Fig).

A post-hoc ANOVA analysis shows 83% (partial ETA squared) of the variation of the density values reported is explained by the electrical stimulation intensity in the complete data set. Additionally, the observed power is 1.0, suggesting a probability of a type II error is less than 0.00. Furthermore, 62.4% (partial ETA squared) of the variation of the density values reported is explained by the electrical stimulation intensity in the tingling data sub-set, with an observed power of 0.997. Thus, these results suggest the sample size was sufficient.

### Pain descriptors associated with the electrical stimulation intensities

**Fig 4:**
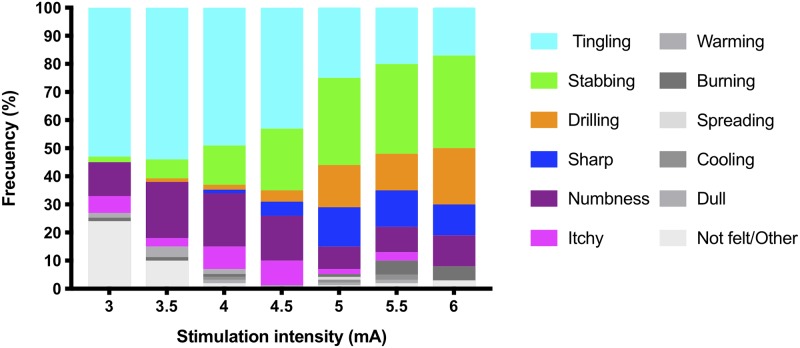
Frequency of descriptors in response to the electrical stimulations. Tingling was most common at lower and stabbing, drilling, and sharp, more common at higher intensities.

Tingling, stabbing, numbness, drilling, and sharp were reported most frequently. Lower electrical stimulation intensities (3 to 4.5mA) were associated with tingling. However, at 4.5mA, a change in pain descriptor frequency was seen (Fig 4), where stabbing, drilling and sharp became more prominent, and reports of tingling reduce. No difference in density values or perceived intensity ratings were found among pain descriptors at the 4mA and 4.5mA stimulation intensity (*P*>0.05).

The Hosmer and Lemeshow test in the logistic regression analyses shows the model fits the complete data set (χ^2^(8) = 5.019, *P* = 0.756). The model explained 10.7% (Nagelkerke R^2^) of the variance in the reporting of tingling and correctly classified 64.1% of the cases. The lower electrical stimulation intensities were associated with the descriptor tingling (B = -0.61, *P*< 0.001). No association was found between the tingling descriptor and the electrical stimulation intensity irrespective of whether the stimulation was applied for the first time or after repeated stimuli (*P*> 0.05).

### Feedback on the usability and tingling motion graphic

Three key themes emerged from the semi-structured interviews: appropriateness of the motion graphic, the usability of Animate Pain, and suggestions for improvement of the tingling graphic (Table 3).

**Table 3:** Summary of comments from the participants.

**Appropriateness of the motion graphic**
"Not happy with the speed parameter. It didn't fit"
"The graphic represents fine the sensation I felt (tingling), but if I see that out of context, I wouldn't think of tingling"
"Neither graphic or canvas matched the sensation"
"The animation represents well the perception"
"Good baseline animation"
**Usability of Animate Pain**
"The speed was confusing and difficult to adjust"
"The name speed is not appropriate and is confusing"
"Not user friendly"
"Liked the 3D image of the hand"
"Difficult to put the sensation to the image"
"The animation represented well the sensation"
"Easy to adjust"
"It's fine. Works well"
**Suggestions for improvement**
"It should have been an image of the whole hand"
"I would have liked to be able to change (the shape of the dotted animated graphic) from dots to sharp”
"Difficult to see with gray on gray"

#### Appropiateness of the motion graphic

Overall, participants had positive impressions with the use of the tingling motion graphic as a visual representation of the evoked sensations (*“Good baseline graphic”*, *“The graphic matched the sensation”*). Interestingly, many highlighted that the motion graphic did not accurately represent sensations other than tingling.

#### Usability of animate pain

Participants reported difficulty adjusting the speed feature and explained the term *“speed”* was inappropriate, and the adjustment of the speed VAS was not intuitive. On the other hand, the density VAS was reportedly intuitive and easy to adjust. The 3D-image of the hand was well received. However, participants expressed difficulty visualizing the grey dots of the motion graphic over the light grey coloured hand image.

#### Suggestions for improvement

Participants suggested displaying an image of the dorsal and ventral aspects of the hand, as three participants perceived sensations in the dorsal aspect of the hand or finger, but were not able to capture them. Furthermore, a suggestion to add a feature that allows the transition of the dots to a new motion graphic shape when the sensations changed from tingling to stabbing or sharp.

## Discussion

This study assessed the relationship between an embodied sensory experience and the ability to translate the perception of this experience visually using modifiable motion graphics.

Participants perceived differences in the intensity of the tingling sensation elicited by a range of electrical stimulations intensities and adjusted the motion graphic systematically. Participants' adjustment behaviour shows the number of dots (density) that appear and disappear at any given time in the motion graphic correlates to the intensity and perceived intensity of the electrically evoked sensation. Furthermore, many participants selected the tingling descriptor to describe the electrically evoked sensation, and semi-structured interviews confirmed that the motion graphic depicted a tingling sensation. An intriguing finding is stabbing was most frequently reported following tingling with increasing and higher electrical stimulation intensities.

### Electrical stimulation as an experimental model of tingling

Previous studies exploring the psychophysics of pain qualities have also evoked similar tingling sensations using electrical stimulations [58–61] and rated the evoked perceptions using the INDSCAL model. In the INDSCAL model, for example, tingling is described as being between the qualities of comfortable and moderate and evoked in the low range (13-30mW) of electrical stimulation intensities [58,59]. In our study, the tingling was also most frequently selected at the lower range of electrical stimulation intensities. This finding suggests that the stimulation protocol served as an appropriate experimental model of tingling in the range of 3 to 4.5mA.

Results from the logistic regression indicate no habituation or adaptation effect, and thus, the number of stimuli and inter-stimulus interval unlikely influenced the pain quality selection or the associated modification of the motion graphic. The similar results of the post-hoc graphical trend analysis of the first three random stimulations and the tingling data sub-set as compared to the complete data set, indicated no learning, habituation, or adaptation effects to the electrical stimulations as the experimental session progressed. Moreover, based on these graphical trends, the density default setting at the study onset (0.5 dots per random unit) corresponded to the range of 4-5mA. Thus, the density default setting may not have influenced the adjustment of the density modifier.

### Pain descriptors and electrical stimulation intensity

The results of the current study show a clear transition in the electrically evoked sensations on the fingertip from tingling to a stabbing, and then to drilling or sharp descriptors as the intensity of the electrical stimulations increases. A consistent reduction in the reports of tingling coincides with a simultaneous increase in stabbing, drilling, and sharp descriptors, where tingling precedes more intense sensations.[13,38] Moreover, the transition from one pain quality descriptor (tingling) to the others (stabbing, drilling, and sharp) may explain the drop in the perceived intensity ratings with increasing electrical stimulation intensity, as evident when assessing the tingling data sub-set. This drop of intensity rating may reduce the correlation found in this study between the perceived intensity ratings and electrical stimulation intensity.

A hierarchy of qualitative descriptors was first proposed in the MPQ [13,38], where pain descriptors were sub-grouped into 11 different categories according to the similarity of the sensation being described. For example, the descriptors burning, hot, scalding, and searing belong to the same category. Additionally, in the short-form MPQ, the descriptors within each section are ranked on a categorical scale of 0 “none”, 1 “mild”, 2 “moderate”, and 3 “severe”, creating a rank-order of the descriptor’s intensity. Furthermore, the short-form MPQ-2 [26] modified the descriptors’ scale ranking from 0 “none” to 10 “worst imaginable”. Other studies [27,62,63] have also explored the relationship between pain descriptors and the stimulus intensity, and suggest that pain descriptors can be ranked based on the stimulation intensity. This ranking is called multidimensional scaling of pain and indicates that there is a transition from one pain descriptor to the next descriptor in the rank [58,60,64,65]. This multidimensional scaling of pain can be used to assist a mechanism-based diagnosis such as neuropathic [30,66] and cancer pain [67].

By developing a motion graphic depicting increasing intensities of, for example, tingling, burning, or itching, one may be able to quantify changes in the sensory dimension of pain. In this present study, the range to control the density parameter in the motion graphic did not reach a maximum. Further increases in density did not reach beyond 5.0 mA, and the electrically evoked stimulations transitioned from tingling to non-tingling sensations before 5.0 mA. Together the results suggest an upper limit in density for the tingling motion graphic exists, and this limit may reflect a critical transition point for altering the shape language to depict stabbing. Moreover, the similarities in individual adjustments to the range of electrical stimulation intensities suggest a universal perception and visual correlate may exist for tingling. Whether this is true for other pain quality descriptors is unknown. In support of these adjustment behaviour results, the semi-structured interviews revealed the tingling graphic as appropriate and clarified the need to alter the shape of the dots associated with higher electrical stimulation intensities to reflect a stabbing sensation.

### The role of modifiable motion graphics in pain assessment

In this study, the motion graphic design intended to depict the sensations evoked by a range of electrical stimulation intensities. The software application enabled participants to modify the motion graphic to match changes in perception using adjustable density and speed VAS. The density as compared to speed VAS was utilized more frequently and was altered across a range of electrical simulations. These results suggest that the density parameter of a motion graphic depicting tingling may be a quantifiable outcome. A future application of modifiable motion graphics with adjustable parameters can be used to probe differences or track changes in health conditions or status, such as in neuropathies.

Further modifiable motion graphics can be used to explore and achieve a better understanding of the psychophysics of pain. Modifiable motion graphics provide a means to capture and quantify changes in perception that go beyond a traditional pain VAS. A deeper understanding of the psychophysics of pain perception may have a decisive role in the interpretation of pain processing [68]. Interestingly, motion graphics may have the additional advantage of overcoming the ambiguity in pain descriptors’ meaning and understanding, cultural background, limited language skills, and cognitive impairment [27,45]. Additionally, a solution to understand better the neurophysiological underpinnings that associate with the transition from one pain quality to another would be to incorporate a function enabling a change in the shape of the motion graphic elements, and in this study, the dot for tingling.

A limitation of this study was the lack of options for choosing more than one motion graphic, thus limiting the participants’ options of digitally expressing alternative sensations. Another limitation was that even though all participants were professionally fluent in English, some had English as a second language. Thus, a language influence on the findings related to the pain descriptor selection cannot be excluded. A third limitation may be that only the glabrous aspect of the hand was shown during the recording. The focus group interviewed revealed that some participants perceived a sensation in the dorsal aspect of the hand. Thus the area associated may be underestimated and, therefore, this could influence the relation reported between area and stimulation intensity. However, results suggest that the area did not spread distally with the increase in electrical stimulation and, perhaps, the evoked perception spread deeper. A fourth limitation was the usability issues with the speed modifier, as reported by the participants during the semi-structured interviews.

Enabling a real-time adjustment of a motion graphic is a novel approach creating new possibilities for understanding perception. Such adjustment features would also add quantifiable data making comparisons easier among different population cohorts. Additionally, in conditions where tingling may transition to stabbing or sharp, for example, such changes may indicate a worsening of the symptoms. Therefore, additional measures that can quantify the change in the quality of symptoms, such as the motion graphic used in this study, may be useful for monitoring health status. These findings underline the importance of evaluating, not just the pain descriptor of the sensations evoked, but the quantitative changes in the perceived intensity of the evoked sensations as offered by motion graphics.

Furthermore, the development and implementation of motion graphics reflecting burning, stabbing, throbbing, or itching in a clinical setting may remove the limitations of comprehensive language skills and prior experience imposed by pain descriptors. Therefore, motion graphics represent a potentially useful method to visualize and quantify sensory perceptions in research and clinical settings. Children, cognitively impaired, or those with limited working native language skills could directly benefit.

This study investigated a modifiable motion graphic as an approach to capture, identify, and quantify changes in electrically-evoked tingling sensations. The motion graphic tested was perceived to reflect a tingling sensation, and modulation of the motion graphics was strikingly similar across participants over a wide range of electrical stimulation intensities. We conclude that although many descriptors can describe the sensory dimension of pain, there may be a universal embodied experience of these sensations. The next step is to determine the utility of using modifiable motion graphics in communication, clinical assessment, and as a research tool.

## Supporting information

S1 Multimedia file Video showing the VAS adjustments of the tingling motion graphic. (MOV)

S1 Fig Graphical “trend” check. This graphical trend shows the relationships among the density, perceived intensity ratings, and the electrical stimulation intensity in the complete data set, tingling data sub-set, and the three first random stimulations from the complete data set.
(TIFF)
