# Supplementary figures and images for: Modifiable motion graphics for capturing sensations
Source: PLoS One. 2020 Feb 24;15(2):e0229139. doi: 10.1371/journal.pone.0229139 (PMC7039426; doi:10.1371/journal.pone.0229139)

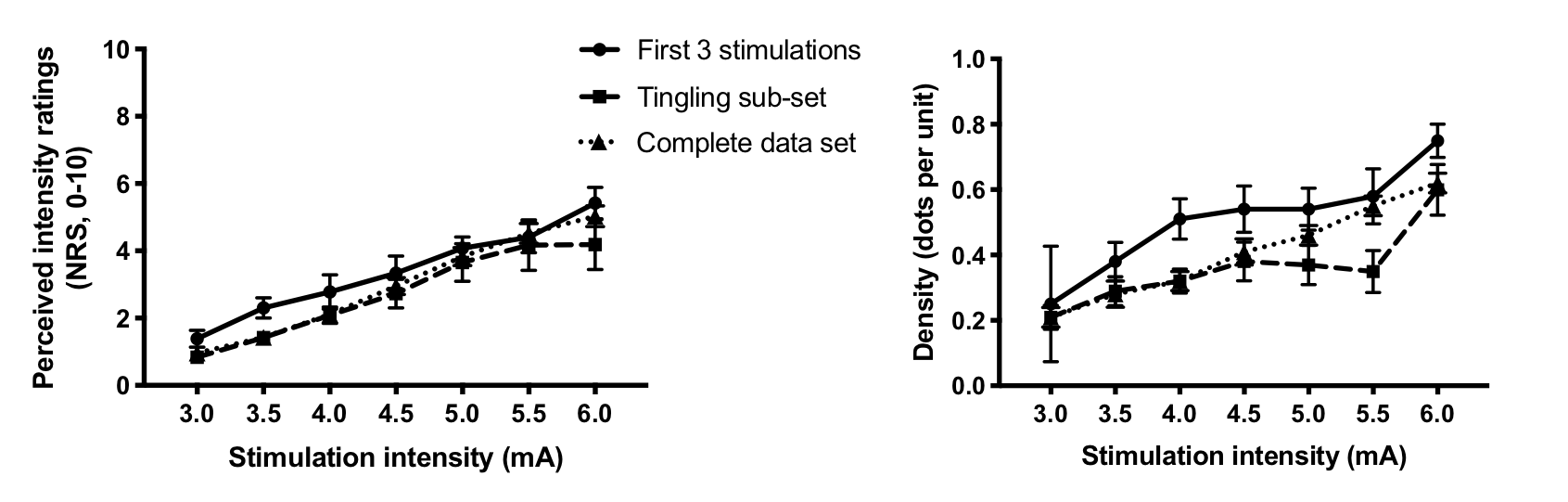

Supplement: S1 Fig — This graphical trend shows the relationships among the density, perceived intensity ratings, and the electrical stimulation intensity in the complete data set, tingling data sub-set, and the three first random stimulations from the complete data set. (TIFF) [file pone.0229139.s002.tiff]
